# Prediction of antimicrobial resistance of *Klebsiella pneumoniae* from genomic data through machine learning

**DOI:** 10.1371/journal.pone.0309333

**Published:** 2024-09-18

**Authors:** Chiara Condorelli, Emanuele Nicitra, Nicolò Musso, Dafne Bongiorno, Stefania Stefani, Lucia Valentina Gambuzza, Vincenza Carchiolo, Mattia Frasca

**Affiliations:** 1 Department of Electrical Electronic and Computer Science Engineering, University of Catania, Catania, Italy; 2 Department of Biomedical and Biotechnological Sciences (Biometec), University of Catania, Catania, Italy; North Carolina State University, UNITED STATES OF AMERICA

## Abstract

Antimicrobials, such as antibiotics or antivirals are medications employed to prevent and treat infectious diseases in humans, animals, and plants. Antimicrobial Resistance occurs when bacteria, viruses, and parasites no longer respond to these medicines. This resistance renders antibiotics and other antimicrobial drugs ineffective, making infections challenging or impossible to treat. This escalation in drug resistance heightens the risk of disease spread, severe illness, disability, and mortality. With datasets now containing hundreds or even thousands of pathogen genomes, machine learning techniques are on the rise for predicting antibiotic resistance in pathogens, prediction based on gene content and genome composition. Aim of this work is to combine and incorporate machine learning methods on bacterial genomic data to predict antimicrobial resistance, we will focus on the case of *Klebsiella pneumoniae* in order to support clinicians in selecting appropriate therapy.

## Introduction

When microorganisms, such as bacteria, viruses, fungi, and parasites, adapt and grow in the presence of medications, they can develop resistance to such medications, that once affected them. This phenomenon, known as antimicrobial resistance (AMR), refers to bacteria ability to survive after exposure to a specified concentration of antimicrobial substances [1–3]. Bacterial resistance can be a natural property or a secondarily acquired mechanism. Surviving antibiotic effects is a normal bacterial reaction, leading to the creation of a clone capable of withstanding the antibiotic [4]. Although AMR is a common mechanism, its specific characteristics depend on the bacterial species, the antibiotics involved and the distribution of resistant strains in different contexts (e.g. hospitals, communities, animal husbandry). Resistant strains are classified by genus, species and antibiotic resistance phenotype [5]. This phenotype is established by comparing the list of antibiotics active on the reference strain with those the tested strain is resistant to, representing acquired resistance.

AMR is a significant threat to global public health [6, 7]. Physicians have several reasons to be concerned about bacterial resistance, as resistant bacteria, notably *Staphylococcus aureus*, *Enterococcus spp.*, *Klebsiella pneumoniae*, and *Pseudomonas aeruginosa*, are becoming increasingly prevalent in healthcare institutions [8]. The inability to treat infectious diseases with antibiotics raises concerns for the future of healthcare, leading to serious illnesses, prolonged hospital admissions, increased healthcare costs, and treatment failures [9]. AMR compromises the ability of the immune system to defeat infectious diseases, affecting vulnerable patients undergoing various medical treatments. Individuals with chronic conditions like diabetes, asthma, rheumatoid arthritis and cystic fibrosis are also heavily impacted [10, 11].

Identifying strains resistant to antibiotics is crucial for reducing drug abuse, but traditional methods are often time-consuming. Recent studies, such as those in [12], highlight the effectiveness of machine learning (ML) methods in predicting AMR across various bacterial strains. ML algorithms, including support vector machines, logistic regression models, and random forests, have demonstrated high accuracy in predicting AMR mechanisms like efflux pumps, target modifications, and enzymatic inactivation. These algorithms, when trained on whole-genome sequencing, offer a time-efficient alternative to traditional culturing studies [13, 14]. ML algorithms have also proved to be effective in predicting new antibiotics, AMR genes, and AMR peptides [15, 16]. Overall, these algorithms provide a promising avenue for swiftly and precisely addressing AMR (for an in-depth technical review of recent studies using ML-driven solutions to solve the AMR problem see [17]).

However, despite the availability of substantial data, a gap still persists in applying predictive modeling to support antibiotic prescription decisions, highlighting the urgent need for an evidence-based framework in clinical decision support systems for antimicrobial management. Recent literature highlights attempts to use ML algorithms to enhance antibiotic prescribing practices. A recent study [18–20] for instance demonstrates that off-the-shelf ML algorithms, trained on a relatively small dataset, provide informed predictions on antibiotic susceptibilities, surpassing traditional logistic regression models. The Random Forest method emerges as a top performer, with input features such as time (from admission to blood culture), patient age, and infection acquisition source being crucial predictors. The study underscores the potential of ML in improving antibiotic prescribing practices without extensive data pre-processing or algorithmic modification. An in-depth exploration of ML methods applied to AMR datasets obtained through mass spectrometry is reported in [21].

Aim of our work is to classify bacteria strains as resistant or susceptible by using ML techniques, ultimately predicting resistance from genomic data in a rapid and accurate way. The same goal was pursued in other papers, but targeting other bacterial species such as Meticilllin resistant *Staphylococcus aureus* (MRSA), *Enterococcus spp.* or *Escherichia coli*. For example, in [22] ML methods have been used for prediction of AMR developed by *Escherichia coli* in response to four different antibiotics. Another study on the *E. coli* applied ML models to predict resistance from the whole genome sequences [13]. Similar studies were also conducted on the *Staphylococcus aureus* [23–25] or on the *Streptococcus pneumoniae*, just to name a few examples. This work delineates a promising path in the predictive field that hopefully can also be applied to clinical practice [26].

**Fig 1:**
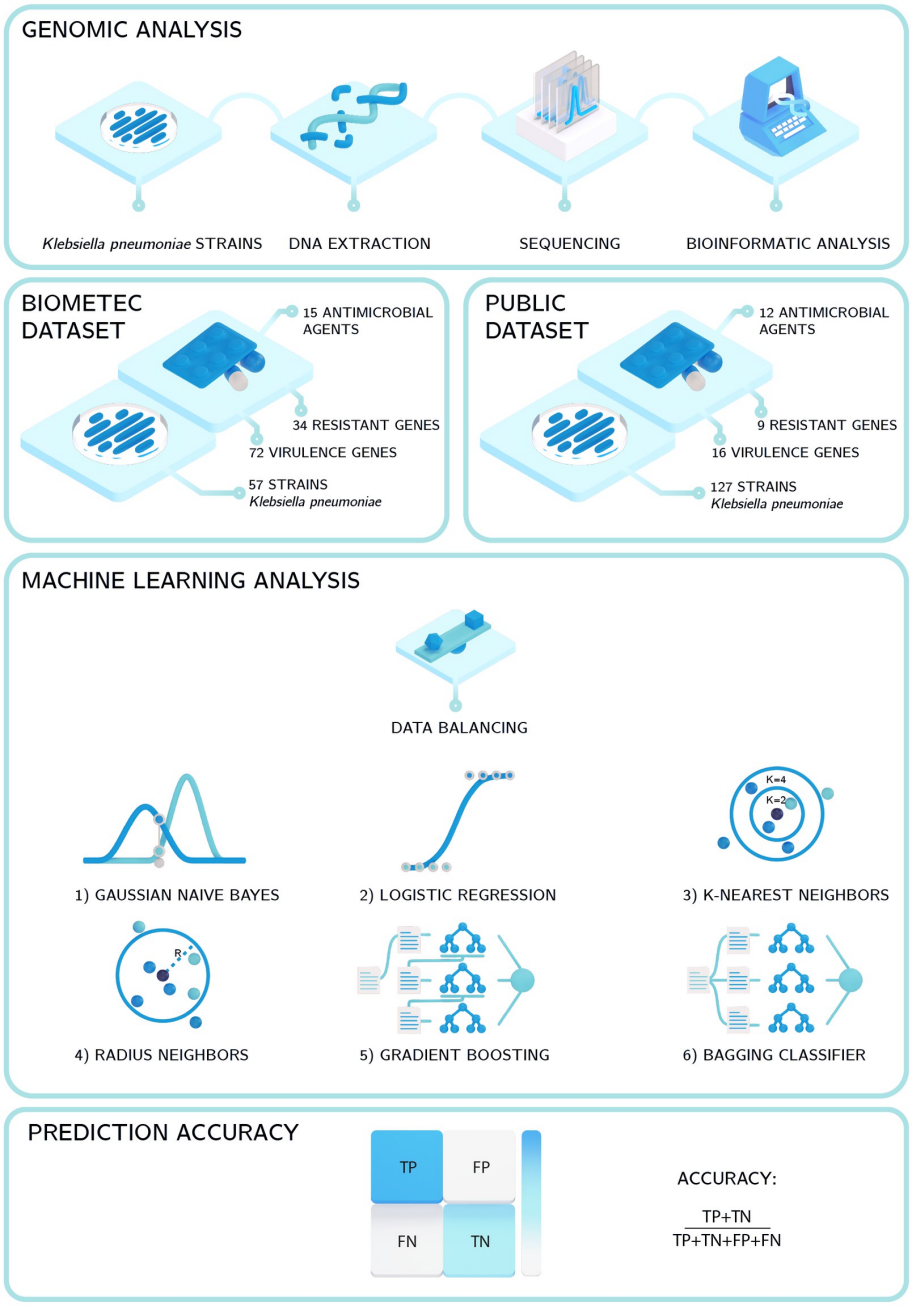
Overview of the approach. Overview of the approach used for predicting resistance/susceptibility to an antimicrobial agent of a strain from genomic data. We start from genomic data contained in two datasets, one derived in the framework of this study and a public one [30]. These data are the result of genomic analysis techniques, including Next-Generation Sequencing (NGS). The data obtained (resistance or susceptibility of bacterial strains to different antibiotics, and presence or absence of resistance/virulence genes for each strain) are then provided as input to six different ML algorithms whose outcome is a binary value predicting the resistance or susceptibility of the strain to the antimicrobial agent.

In this paper, we focus on *Klebsiella pneumoniae*. Other studies performed on the same bacterium and using ML analysis, have considered prediction of the phenotypic polymyxin resistance [27], the identification of the resistance features starting from whole-genome single-nucleotide polymorphism data [28], or multilabel classification of AMR with missing labels [29]. Here we deal with the problem of predicting the resistance of *Klebsiella pneumoniae* to several antibiotics from genomic data containing only a subset of all possible resistance and virulence genes. We perform the analysis on two datasets differing in the size of the sample, the genes considered and the origin of the bacteria, in order to study how the different features of the datasets impact the accuracy of the prediction. An overview of the approach adopted in our work is shown in Fig 1. Our analysis starts from the genomic data of the two different datasets containing information on AMR of several strains of *Klebsiella pneumoniae*. These data are the results of DNA extraction, sequencing and bioinformatic analysis extracting resistance and virulence genes of the strains. The data provide the input to ML analysis that, through several steps, provides a classification of bacteria strains as resistant or susceptible to a series of different antimicrobials.

## Materials and methods

### Dataset description

In our study we use genomic data to predict the antibiotic resistance of *Klebsiella pneumoniae*. Genomic sequences of bacteria are in fact essential for understanding the molecular mechanisms underlying their resistance to antibiotics. The data include information about the DNA, genes involved in resistance, and genetic mutations. The data encompass details regarding the presence or absence of specific resistance determinants. Since the paper focuses on *Klebsiella pneumoniae*, we pay particular attention to genes that provide resistance to *β*-lactam antibiotics, such as carbapenemases and extended-spectrum *β*-lactamases. This is crucial in the clinical domain due to the alarming rise in resistance, particularly in nosocomial settings, to the above mentioned antibiotics [31]. The quest for genes responsible for these resistances involves the use of whole genome sequencing through Next Generation Sequencing (NGS) [32].

Here we consider two datasets comprising the genomic data of *Klebsiella pneumoniae* strains. The first dataset contains the data collected by our research group at the Department of Biomedical and Biotechnological Science (Biometec). For simplicity, we will refer to this dataset as the Biometec dataset. The second is a public dataset, analysed in [30].

The Biometec dataset contains the genomic data of 57 strains of *Klebsiella pneumoniae*. For each strain, the dataset has information on the resistance/susceptibility to 15 antimicrobial agents and the presence/absence of 34 resistance genes, divided into 9 categories as follows: *β*-lactamase, quinolones, aminoglycosides, fosfomycin, sulfonamide, phenicols, macrolides, tetracicline and others, as illustrated in Table 1. For the majority of the strains (50 out of 57), the data also contains information on virulence genes. Specifically, the dataset includes information on the presence or absence of 78 virulence genes. The strains have different sources; in particular, we have considered strains taken from blood, urine, respiratory tract, rectal swabs and one strain from a burn swab. The samples included in the study were part of a strain collection of the laboratory of Microbiology at Biometec. The strains were collected in different hospitals located in South Italy during May 2020 and July 2023. For each patient a single strain was included in the dataset. Identification and antimicrobial susceptibility were previously performed by the VITEK 2^®^ system (bioMerieux, Marcy l’Etoile, France) at the hospitals and re-confirmed by standard methods (EUCAST, 2024). VITEK 2^®^ is a fully automated system that performs bacterial identification and antibiotic susceptibility testing. It uses Advanced Colorimetry™, an identification technology that enables identification of routine clinical isolates. Advanced Colorimetry provides high discrimination between species and low rate of multiple choice and misidentified species. Breakpoints of antibiotics for the interpretative criteria for clinical isolates were used according to the EUCAST v 14.0 (EUCAST, 2024).

**Table 1:** List of genes included in the Biometec dataset divided into nine categories: *β*-lactamase, quinolones, aminoglycosides, fosfomycin, sulfonamide, phenicols, macrolides, tetracicline and ‘others’.

Type	Genes
*β*-***lactamase***	*bla*_SHV-28_, *bla*_SHV-106_, *bla*_SHV-187_, *bla*_SHV-205_, *bla*_SHV-212_, *bla*_KPC-3_, *bla*_KPC-31_, *bla*_KPC-34_, *bla*_OXA-1_, *bla*_OXA-9_, *bla*_TEM-181_, *bla*_CTX-M-15_
*Quinolones*	*qnrB17*
*Aminoglycosides*	*aph(3”)-Ib, aph(3”)-Ib10, aph(6)-Id*, *aac(3)-IIe*, *aac(6’)-Ib-cr6*, *aac(6’)-Ib10, aadA2*, *ant(2”)-Ia*, *ant(3”)-IIa*, *armA*
*Fosfomycin*	*fosA6*
*Sulfonamide*	*sul1, sul2, dfrA12, dfrA14, dfrA17*
*Phenicols*	*catB3, catI*
*Macrolides*	*mphE*
*Tetraciclin*	*tetA*
*Others*	*qacEdelta1*

The second dataset (i.e. the public one) contains the genomic data of 127 *Klebsiella pneumoniae* strains taken from four different Catalonian hospitals collected over six months in 2016. Also in this case the strains are divided into three different categories, based on the origin of the bacterium: strains taken from blood, urine and the respiratory tract. The dataset includes the following information: resistance/susceptibility of each strain to 12 antimicrobial agents; presence/absence of 9 resistance genes for each strain and presence/absence of 16 virulence genes for each strain. As discussed in detail in the next sections, in our study we mainly concentrated on data pertaining to resistance genes. Specifically, the nine resistance genes appearing in the dataset are reported in Table 2.

**Table 2:** List of resistance genes included in the public dataset divided in four categories: *β*-lactamase, quinolones, aminoglycosides and sulfonamide.

Type	Genes
*β*-***lactamase***	*bla*_SHV-1_, *bla*_TEM-1_, *bla*_CTX-M1_, *bla*_OXA-48_
*Quinolones*	*qnrB*
*Aminoglycosides*	*aac(6*’)-Ib-cr, aadb
*Sulfonamide*	*sul1, sul2*

The main features of the Biometec and public datasets are summarized, respectively, in Table 3 and in Table 4, where we have reported the resistant and susceptible strains for the antimicrobial agents considered in our study.

**Table 3:** Summary of the main features of the Biometec dataset. The following antimicrobial agents included in the dataset have been considered in our study: *amoxicillin clavulanic acid* (AMC), *cefepime* (FEP), *ceftazidime* (CAZ), *imipenem* (IMI), *aztreonam* (AZT), *ceftazidime/avibactam* (CZA), *meropenem* (MEM), *meropenem/vaborbactam* (MEM/VAB), *ciprofloxacin* (CIP), *gentamicin* (CN), *amikacin* (AK), *colistin* (COL), *fosmomycin* (FOS), *trimethoprim-sulfamethoxazole* (SXT). The total number of strains is 57, divided between resistant and susceptible.

Antimicrobial agent	Resistant strains	Susceptible strains	Type
**AMC**	57	0	*β*-*lactamase*
**FEP**	57	0	
**CAZ**	57	0	
**IMI**	41	16	
**AZT**	55	2	
**CZA**	43	14	
**MEM**	44	13	
**MEM/VAB**	16	41	
**CIP**	57	0	*Fluorochinolone*
**CN**	54	3	*Aminoglycoside*
**AK**	45	12	
**COL**	13	44	*Colistin*
**FOS**	40	17	*Fosfomycin*
**SXT**	39	18	*Sulfonamide*

**Table 4:** Summary of the main features of the public dataset. The following antimicrobial agents included in the dataset have been considered in our study: *amoxicillin clavulanic acid* (AMC), *cefepime* (FEP), *ceftazidime* (CAZ), *imipenem* (IMI), *aztreonam* (AZT), *ciprofloxacin* (CIP), *gentamicin* (CN), *colistin* (COL), *fosfomycin* (FOS), *trimethoprim-sultamethoxazole* (SXT). The total number of strains is 127, divided between resistant and susceptible.

Antimicrobial agent	Resistant strains	Susceptible strains	Type
**AMC**	50	77	*β*-*lactamase*
**FEP**	30	97	
**CAZ**	47	80	
**IMI**	14	113	
**AZT**	37	90	
**CIP**	52	75	*Fluorochinolone*
**CN**	21	106	*Aminoglycoside*
**COL**	2	125	*Colistin*
**FOS**	18	109	*Fosfomycin*
**SXT**	44	83	*Sulfonamide*

The two datasets have the following ten common antimicrobial agents:*Amoxicillin/clavulanate* (AMC), *Cefepime* (FEP),*Ceftazidime* (CAZ), *Imipenem* (IMI),*Aztreonam* (AZT), *Ciprofloxacin* (CIP),*Gentamicin* (CN), *Colistin* (COL), *Fosfomycin* (FOS), *Trimethoprim/sulfamethoxazole* (SXT). The comparison between the results that can be obtained from the two datasets has been carried out taking into account these agents. However, given their clinical importance, in our study we have also considered four other antimicrobial agents that are only present in the Biometec dataset, but not in the public dataset. These are: *Ceftazidime/Avibactam* (CZA), *Meropenem* (MEM), *Meropenem/Vaborbactam* (MEM/VAB) and *Amikacin* (AK). In fact, *Ceftazidime*, a 3rd generation cephalosporin, is an important antibiotic molecule used in clinical practice. This antibiotic is used in combination with *Avibactam*, an inhibitor of class A, C and some class D *β*-lactamase. The antibacterial spectrum of *Ceftazidime-Avibactam* covers >99% of enterobacteria, including strains carrying extended-spectrum *β*-lactamase [33], such as *Klebsiella pneumoniae*, under examination in this study. The other three antibiotics play a prominent role in clinical practice, in particular *Meropenem* (MEM), is a *β*-lactam antibiotic belonging to broad-spectrum carbapenems, only and in combination with the *β*-lactamase inhibitor *Vaborbactam* (MEM/VAB) and *Amikacin* (AK), a semi-syntetic aminoglycoside antibiotic used for most resistant Gram-negative bacteria. Due to their activity against multidrug resistant (MDR) pathogens, all aforementioned antibiotics should be considered as some of the most important molecules in case of infections supported by MDR pathogens.

### Genomic analysis

Genomic analysis of bacteria is a complex and detailed process to identify their genetic code. DNA sequencing was performed by means of a new technology called Next-Generation Sequencing (NGS) [32]. This technology allows large numbers of genes to be sequenced in short periods of time. The process begins with the extraction of DNA from the bacterium of interest, which can be done from bacterial cultures or samples. The extracted DNA is then split into small segments which are then treated with different techniques before moving to the sequencing phase. The sequencing phase occurs in parallel and is done using techniques based on different principles. In our case Illumina^®^ (fluorescent sequencing, short reads) was used. The sequences generated by NGS are then analyzed to search for genes with specific characteristics, such as antibiotic resistance or virulence.

Genomic analysis was performed on strains collected at the laboratory of Medical Molecular Microbiology and Antibiotic Resistance (MMAR) at Biometec, according to the three steps that are discussed in the following.

#### DNA extraction

The first step is DNA extraction from the sample of interest. DNA extraction was carried out following the manufacturer’s instructions provided by QIAGEN QIAamp^®^ DNA Mini Kit (Ref. 51304, QIAGEN, 40724 Hilden, Germany). DNA was quantified using both the Eppendorf BioPhotometer^®^ D30 and the fluorimeter Qubit dsDNA BR Assay Kit to evaluate purity and quantity of the initial sample, respectively (Ref. 32850, Invitrogen, 92008 Carlsbad, CA, USA) [34].

#### Next Generation Sequencing (NGS)

A concentration of 100*ng* of each sample was used for NGS. This step was performed in the laboratory of Molecular Biology at the University of Catania on an Illumina^®^ MiSeq platform according to the manufacturer’s instructions provided in Watchmaker DNA Library Prep kit with Fragmentation–Watchmaker Genomics^®^ (Ref. 7K0013–024, 5744 Central Avenue, Suite 100 Boulder, CO 80301, USA). Indexes were provided with Twist Universal Adapter System (16 Indexes, 16 Samples) (Ref. 101307, Twist Bioscience, HQ 681 Gateway Blvd, South San Francisco, CA 94080FAQ). Libraries were quantified and their quality was evaluated using both the fluorometric Qubit dsDNA HS Assay Kit (Ref. Q32851, Invitrogen, Carlsbad, CA 92008, USA) and the Agilent^®^ High Sensitivity DNA Kit (Ref. 5067–4626). Denature and dilute libraries were performed following the “Denature and Dilute Libraries Guide” protocol provided by Illumina^®^, choosing 8,5 pM as the loading concentration. Finally, sequencing was performed using the MiSeq Reagent Kits v3 (Ref. 15043895, Illumina, Inc., 92122, San Diego, CA, USA). The Sample Sheet was created using the Local Run Manager v3 software, and following the instructions in the Local Run Manager v3 Software Guide provided by Illumina^®^. [34]

#### Bioinformatic analysis

Data were analyzed using the QIAGEN CLC Genomics Workbench software and following the user manual for the CLC Microbial Genomics Module 23.0.2, released on July 7, 2023 (QIAGEN, Aarhus, 8000 Denmark), to assign resistance gene, virulence and Multi-locus Sequence Type (MLST).

### Preprocessing of the datasets

#### Correlation between data

From the public database described above we extract a smaller data set by considering the following preliminary analysis. First of all, we perform a correlation analysis aiming at reducing the number of input data for the application of the ML methods. To this aim we calculated the Pearson correlation coefficient [35], by using MATLAB^®^.

The correlation coefficient of two random variables is a measure of their linear dependence. Assumed that each variable has *N* scalar observations, then the Pearson correlation coefficient *ρ* is defined as [36]:

$$\begin{array}{c} \rho \left(A,B\right)=\frac{1}{N-1}\sum_{i=1}^{N}\left(\frac{A_{i}-\mu_{A}}{\sigma_{A}}\right)\left(\frac{B_{i}-\mu_{B}}{\sigma_{B}}\right) \end{array}$$ (1)

where *μ*_*A*_ and *σ*_*A*_ are the mean and standard deviation of the variable *A*, respectively, and *μ*_*B*_ and *σ*_*B*_ are the mean and standard deviation of *B*. We can also define the correlation coefficient in terms of the covariance of *A* and *B*:

$$\begin{array}{c} \rho \left(A,B\right)=\frac{cov(A,B)}{\sigma_{A}\sigma_{B}}. \end{array}$$ (2)

**Fig 2:**
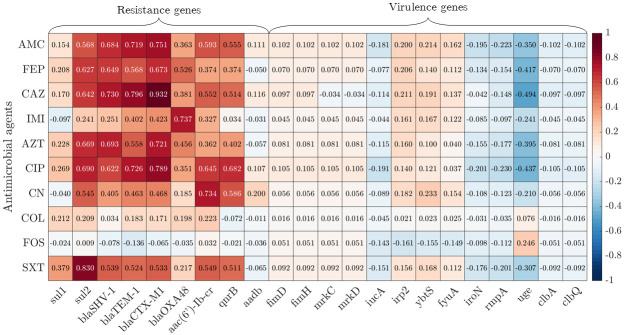
Pearson correlation coefficient. Values of the Pearson correlation coefficient *ρ* obtained for the public dataset. We find a strong correlation (|*ρ*|>0.8) between resistance genes (*sul1*, *sul2*, *bla*_SHV-1_, *bla*_TEM-1_, *bla*_CTX-M1_, *bla*_OXA-48_, *aac(6’)-Ib-cr*, *qnrB*, *aadb*) and resistance to antimicrobial agents. Virulence genes are less correlated with resistance to antimicrobial agents, or at most the correlation is negative (the presence of the genes may imply non-resistance to antimicrobial agents). We also notice a low correlation between the genes and the antimicrobial agent FOS. Virulence genes ycfM, entB and wabG are not reported because they are present in all strains (all 1), it means that *σ* = 0 such that *ρ* cannot be evaluated.

The Pearson correlation coefficient *ρ* has been used to evaluate the correlation between the resistance to each antimicrobial agent and the presence of each resistance or virulence gene. The results for the public database are illustrated in Fig 2. Following [37], values of |*ρ*| > 0.8 are considered an indication of a strong correlation, values of 0.4 < |*ρ*|<0.8 a moderate correlation, while values of 0 < |*ρ*| < 0.4 a weak correlation. Consequently, we observe that there is neither strong nor moderate correlation between the resistance to antimicrobial agents and the presence of virulent genes in the public dataset, while there is a strong/moderate correlation between the resistance to antimicrobial agents and the presence of resistance genes. Taking into account these findings, in this study we focused on the data pertaining to resistance genes.

#### Data balancing

One primary challenge associated with the use of ML methods is the potential imbalance in datasets. This imbalance may manifest as an inadequate representation of one class or, conversely, as the presence of one class containing the majority of the samples, and may lead to biased results. Balancing a dataset facilitates model training by preventing bias towards a specific class. Essentially, the model ceases to favor the majority class solely due to its larger presence in the dataset. To this purpose, oversampling, a technique aiming at increasing the number of samples in the minority class until it matches the majority class, can be used. ML methods replicate samples from the underrepresented class, ensuring the model encounters an equal number of samples from each class, reducing the potential for bias towards the majority class. To address overfitting concerns, ML methods can augment the data, introducing variations to the copies of samples in the minority class compared to the originals.

An extreme scenario of unbalancing is the case where all data belong to the same class. In our case, this has been addressed prior to the application of ML techniques, by excluding data pertaining to antibiotics, where all strains were identified as either resistant or sensitive. For the remaining data, resampling techniques were employed to address any imbalance. Specifically, to this aim, we used the Synthetic Minority Oversampling Technique (SMOTE) [38].

### Machine learning analysis

ML, a branch of artificial intelligence, involves statistical methods for the derivation of models and algorithms enabling computers to analyze and interpret data, recognize patterns, and make decisions or predictions, by the identification of the inherent relationships within data [39]. While traditional models are based on contextual knowledge and experience to address a specific task, ML approaches offer a highly flexible means of constructing models of the relationships between data. To this purpose, different ML methods can be chosen on the basis of the desired outcome, such as, for instance, accuracy, precision and recall.

The typical workflow of ML techniques involves training the model on the learning dataset, evaluating the performance on a given validation dataset and then adjusting the model parameters accordingly. Usually, once the model is trained and tuned, it is then tested on a separate dataset in order to provide a final evaluation of its performance. Learning can be: supervised, when the model is trained on a labeled dataset, making predictions or classifications based on input features; unsupervised, when the algorithm explores data patterns without labeled output, as often used for clustering or dimensionality reduction; or can be based on reinforcement, when agents learn to make decisions by interacting with an environment and receiving feedback in the form of rewards or penalties.

In this work we use supervised learning for classification. This method involves the formalization of the learning process through mathematical functions and optimization, where a mathematical model maps input features to output labels. This mapping is represented by a function, denoted as *f*: *X* → *Y* where *X* is the space of input features and *Y* is the space of output labels. In our research, the initial phase involves data preprocessing (Preprocessing of the datasets).

As already mentioned, for balancing data we used the SMOTE. SMOTE is implemented in Python in the imbalanced-learn library, which can be used in conjunction with scikit-learn. SMOTE is helpful in particular because it balances the class distribution by creating synthetic samples for the minority class by interpolating between samples of the minority class that already exist.

Subsequently, we applied various ML methods. The input sequences used for the AMR classification with ML consist of data indicating the presence or absence of resistance genes in individual bacterial strains. For each strain, we have data to define whether a specific gene was present (denoted as 1) or absent (denoted as 0). As result of the training step, we get the resistance, indicated as 1, or susceptibility, indicated as 0, of the strains to each of the antimicrobial agents considered in our analysis.

The key step necessary for constructing a ML solution, following the pre-processing and preparation of the dataset, is the selection of a suitable model to fit the data. There are diverse ML algorithms in literature and there is no one-size-fits-all approach. The decision on a specific algorithm is often influenced by various factors, including the type, size, and complexity of the data.

In our work six ML models were studied: the Gaussian Naive Bayes, the Logistic regression, the k-nearest neighbors, the radius neighbors, the Gradient Boosting and the Bagging classifier [39].

The code used in our work is written in Python, in particular we used the scikit-learn Python pakage.

The Gaussian Naive Bayes model belongs to a group of supervised learning algorithms known as “Naive Bayes” that use Bayes’ theorem with the “naive” assumption that each pair of features is conditionally independent, given the value of the class variable. The various naive Bayes classifiers differ mainly by their underlying assumptions on the distribution of the likelihood of the features. In case of Gaussian Naive Bayes, it is assumed to be Gaussian. We use the GaussianNB from the package sklearn.naive_bayes of Python, with default parameters.

The second model used is Logistic regression. It is a statistical analysis method for predicting a binary outcome, based on previous observations of a data set. In ML applications, algorithms based on logistic regression allow incoming data to be classified based on historical data. The performance in predicting data classification improves as additional relevant data are used for learning. The logistic regression is implemented in Python in LogisticRegression, from sklearn.linear_model. We use it in “multinomial” multi_class and with a maximum number of iterations equal to 3000.

Two other models we studied are k-nearest neighbors and radius neighbors. They both belong to neighbors-based classification, a type of instance-based or non-generalizing learning. Classification is computed from a simple majority vote of the nearest neighbors of each point. In sklearn.neighbor it is possible to implement the two different nearest neighbors classifiers that we used, KNeighborsClassifier, that implements learning based on the k nearest neighbors of each query point (k integer value), and RadiusNeighborsClassifier, that implements learning based on the number of neighbors within a fixed radius r of each training point (r floating-point value). In both cases we test the models with different parameters and we choose the best solution with the GridSearchCV function, located in sklearn.model_selection. In particular, for the KNeighborsClassifier we set n_neighbors parameter equal to 2,6 or 10, while for weight and algorithm parameters we tested all the possible values (distance and uniform for the weight function used in prediction, and auto, ball_tree, kd_tree and brute for the algorithm used to compute the nearest neighbors). For the other parameters we used default values. For RadiusNeighborsClassifier we set the radius parameter in a range of 9, 30 or 1, while for the weight and algorithm parameters we tested the same values as in the previous case. The other parameters are set by default.

The last two models belong to the class of ensemble methods, where the purpose is to increase generalizability and robustness over a single estimator by combining the predictions of numerous base estimators built with a specific learning algorithm. The goal is to create a powerful ensemble by combining a number of weak models. Typically, ensemble methods belong to one of the two families:*Averaging methods*, where the central idea is to average the results of different estimators working independently of each other (due to its lower variance, the combined estimator typically performs better than any single base estimator); or *Boosting methods*, used when one wants to lessen the bias of the combined estimator by building the base estimators progressively. As an example of the first family, we use the BaggingClassifier and GradientBoostingClassifier from sklearn.ensamble. For BaggingClassifier we use *SVC* (from sklearn.svm) as base estimator. Then we set n_estimator = 10, max_saples = 0.8 and max_fearures = 0.8. As an example of the second family, we use the GradientBoostingClassifier where we set the number of boosting stages equal to n_estimetors = 75, validation_fraction = 0.2, n_inter_no_change = 5, tol = 0.01 and randm_state = 0.

Finally we describe the indicators used to assess the performance of the models. We start from the accuracy, defined as the ratio between the number of correct predictions and the total number of predictions:

$$\begin{array}{c} \text{Accuracy}=\frac{\text{Number}\;\text{of}\;\text{correct}\;\text{prediction}}{\text{Total}\;\text{number}\;\text{of}\;\text{predictions}} \end{array}$$ (3)

We can also compute the accuracy by means of the outcome of the model prediction:

$$\begin{array}{c} \text{Accuracy}=\frac{\text{TP+TN}}{\text{TP+TN+FP+FN}} \end{array}$$ (4)

where TP = True Positive, TN = True Negative, FP = False Positive, FN = False Negative.

The true positive TP outcome is obtained when the model correctly predicts a member of the positive class, i.e., the resistant bacterium is predicted to be resistant. Similarly, a true negative, TN, occurs when the model correctly predicts a member of the negative class, i.e., a susceptible bacterium is predicted to be susceptible. A false positive, FP, is instead obtained when the model incorrectly predicts a member of the positive class, i.e., a susceptible bacterium is predicted to be resistant. Finally, a false negative, FN, occurs when the model incorrectly predicts a member of the negative class, i.e., a resistant bacterium is predicted to be susceptible. FN prediction is the case that requires the most attention, as an incorrect prediction could lead to an ineffective treatment. Notice that the value of the accuracy alone, especially in datasets when the data are unbalanced, may not be very informative, as it does not give information on the number of false positives or false negatives. For this reason, precision and recall parameters can be used:

$$\begin{array}{c} \text{Precision}=\frac{\text{TP}}{\text{TP+FP}} \end{array}$$ (5)

$$\begin{array}{c} \text{Recall}=\frac{\text{TP}}{\text{TP+FN}} \end{array}$$ (6)

In our study, since we use balanced data, the accuracy parameter is a good indicator of the model performance. For this reason, in the following we concentrate on this parameter. However, we note that also the recall parameter may be very significant in our application since it could provide information on the reliability of an antibiotic treatment to patients.

## Results

In this section we discuss the results obtained by applying the ML algorithms previously discussed to the Biometec and public dataset. As discussed in Section Materials and methods, the first step of our analysis was to eliminate the antimicrobial agents associated with bacterial strains that are all resistant or all susceptible to a specific antimicrobial agent. In the Biometec dataset (Table 3) we find that for four antimicrobial agents only resistant strains are available: *Amoxicillin/clavulanate*, *Cefepime*, *Ceftazidime* and *Ciprofloxacin*. Since we cannot predict the resistance or susceptibility for these antimicrobial agents, they have not been considered in the other steps of our analysis.

We first consider the application of the ML methods to the original unbalanced datasets. Using the original datasets, we find that for some antimicrobial agents several of the methods of Section Machine learning analysis fail to converge. In other cases, even if the methods converge, the accuracy values are on average around 10% less, with typically a large number of FP and FN. This confirms the need of balancing for our data.

For this reason, we decided to use a technique for data balancing, in particular SMOTE as discussed in Section Data Balancing. In this way, applying ML methods to data we ensure that learning is possible also for the minority class, which also improves the values of precision, recall and accuracy of the predictions.

Next, we illustrate the results for the balanced data. We anticipate that, comparing the results for all the ML methods of Section Machine learning analysis, the Gradient Boosting classifier and k-nearest neighbors classifier are generally the two methods that better perform, i.e., the two which give the highest accuracy in the prediction.

**Fig 3:**
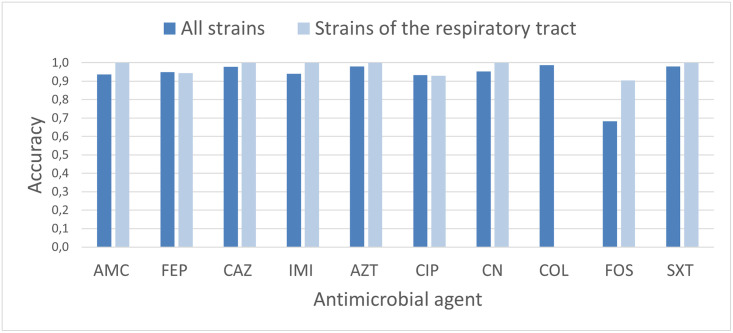
Accuracy values for the public dataset. Comparison of the maximum values of accuracy obtained when using all data of the public dataset or only those on bacterial strains taken from the respiratory tract.

We begin with the analysis carried out on the public dataset. The dataset, as described in Section Dataset description, contains information on the susceptibility or resistance of 127 bacterial strains of *Klebsiella pneumoniae* to 12 different antimicrobial agents. However, as previously discussed, we consider the data only of the 10 antimicrobial agents that are present also in the Biometec dataset. For these agents we apply the six ML methods described in Machine learning analysis and for each of these methods we evaluate the accuracy (4). The results are illustrated in Table 5 and in Fig 3, where they are also compared with the same analysis carried out on a smaller number of strains. In more detail, to test the viability of the method as a function of the size of the analyzed sample, we have applied the same procedure used for the entire dataset to a smaller subset of data. Specifically, we focused the analysis on the 37 strains of the respiratory tract. For the respiratory tract, in fact, all antimicrobial agents, with the only exception of *Colistin* (COL), for which there is a single resistant strain, have a sufficient number of resistant and sensitive strains.

**Table 5:** Maximum values of accuracy obtained with the six ML models for the public dataset. Results are compared in the case when all 127 strains of the dataset or only the 37 strains of the respiratory tract have been considered. For the antimicrobial agent COL in the case of the respiratory tract strains, the accuracy cannot be evaluated since a single resistant strain is available in the dataset.

Antimicrobial	Maximum value of accuracy	
	Whole dataset	Respiratory data
AMC	0,936	1,000
FEP	0,949	0,944
CAZ	0,979	1,000
IMI	0,941	1,000
AZT	0,981	1,000
CIP	0,933	0,929
CN	0,953	1,000
COL	0,987	NA
FOS	0,682	0,905
SXT	0,980	1,000

Table 5 shows that, in the case of the analysis of the whole dataset, the values of accuracy are above 90% for all methods, with the Gradient Boosting classifier performing better than the other techniques for almost all antibacterial agents. The comparison (see also Fig 3) with the accuracy values obtained on the smaller dataset of respiratory strains shows that no clear differences emerge when the whole dataset or a part of it is used for the analysis. We conclude that predicting antimicrobial resistance from genomic data is possible, even when a small number of strains is available, e.g. the subset of the 37 strains related to the respiratory tract bacteria.

Next, we discuss the analysis of the Biometec dataset, which, as described in Section Dataset description, contains information on the resistance or susceptibility to different antimicrobial agents of 57 bacterial strains. The results are illustrated in Table 6, which shows that the highest accuracy values are obtained for k-nearest neighbors ML model, with all other models also displaying good performances (accuracy values greater than 83%).

**Table 6:** Maximum values of accuracy obtained with the six ML models for the Biometec dataset (57 strains).

Antimicrobial	Maximum value of accuracy
IMI	0,920
AZT	0,970
CN	1,000
COL	0,963
FOS	0,833
SXT	0,917

**Fig 4:**
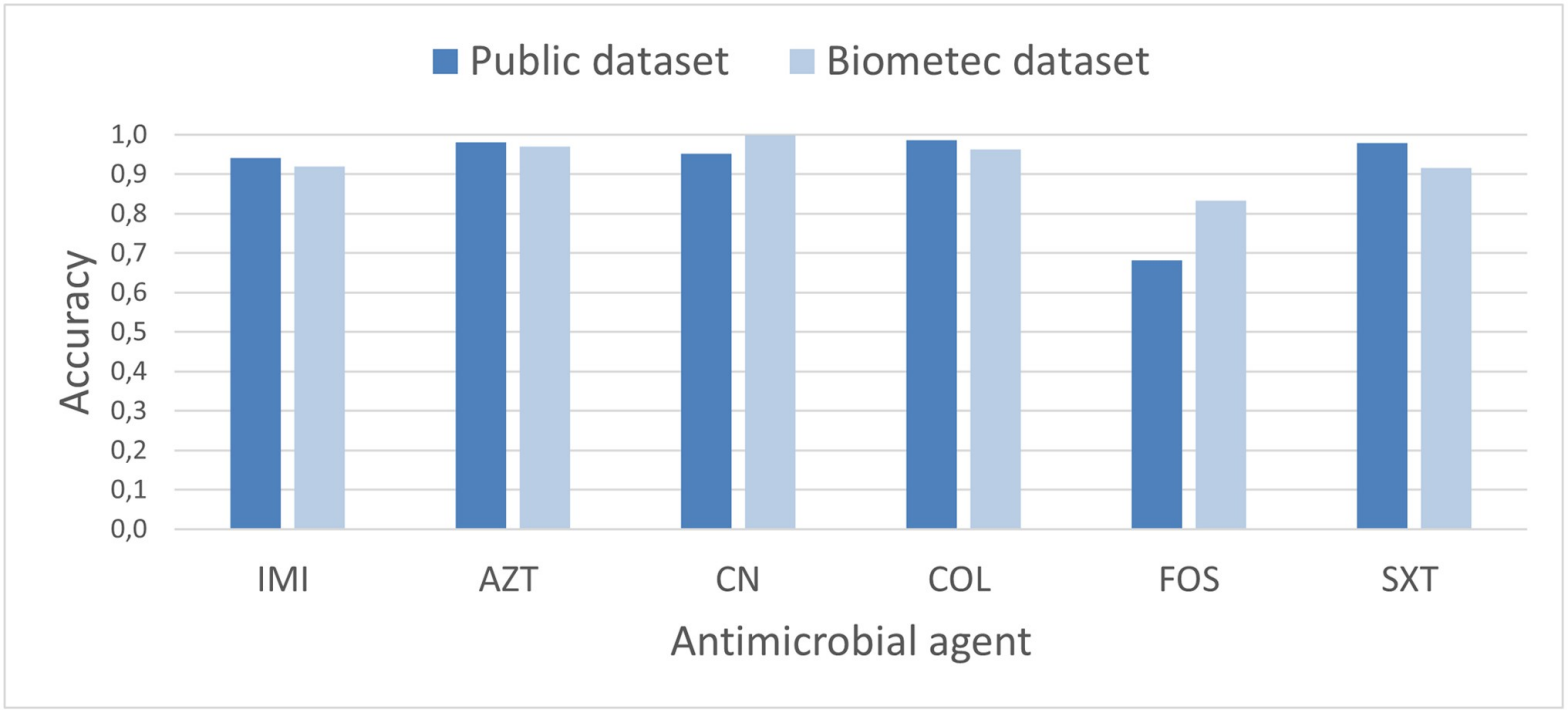
Accuracy values for the public dataset and for the Biometec ones. Comparison between the maximum value of accuracy obtained in the two datasets for six different antimicrobial agents (for the public dataset all 127 strains have been considered).

We then compare the results obtained on the Biometec and the public dataset. In this case, the analysis is carried out on the six antimicrobial agents (IMI, AZT, CN, COL, FOS, and SXT) that are shared by the two datasets. The results are illustrated in Fig 4, where, for each antimicrobial agent, we have reported the maximum value of accuracy obtained by applying the six ML methods to the two datasets. We note a higher value of accuracy for the FOS antimicrobial agent using the Biometec dataset, while for SXT antimicrobial agent a higher value of accuracy is obtained when the public dataset is used. For the other antimicrobial agents similar values of accuracy are obtained in the two datasets. We also notice that, for all antimicrobial agents with the exception of FOS, the accuracy values are close to one.

The result obtained for the FOS antimicrobial agent is particularly interesting. The two datasets we have studied contain different genes. In particular, while the public dataset does not contain any *Fosfomycin*-type gene, the Biometec dataset includes one gene of this type. However, we have found that the presence/absence of this single gene is not enough to explain the different value of accuracy obtained for the two datasets. In fact, removing this gene from the Biometec datasets and applying the six ML methods to this reduced subset of data yields a very similar value of accuracy (in both cases the accuracy is between 0.703 and 0.833, depending on the method used). When Logistic regression, Gradient Boosting and Bagging classifier are used, the same values of accuracy are obtained even if we consider the reduced dataset without the *Fosfomycin*-type gene, e.g. 0.708 for Logistic regression, 0.833 for Gradient Boosting, 0.750 for Bagging classifier. Instead, the accuracy values obtained with the other three methods differ slightly when we do not consider the *Fosfomycin*-type gene among the genes, in particular with Gaussian Naive Bayes we obtained 0.708 and 0.750, with k-nearest neighbors 0.833 and 0.708, with radius neighbors 0.750 and 0.792 respectively in the case of dataset with or without *Fosfomycin*-type gene.

This shows that the analysis performed is far from trivial. The low values of the Pearson correlation coefficient *ρ* observed for this antimicrobial agent (see Fig 2) also suggest that other genes may be needed to predict resistance/susceptibility to this antimicrobial agent.

As discussed in Section Preprocessing of the datasets, the analysis based on the Pearson correlation coefficient reveals a weak correlation between virulence genes and resistance to antimicrobial agents. For this reason, the analysis illustrated so far has been carried out only considering the data related to resistance genes. Here, we shortly comment on the results that are obtained by also including virulence genes in our analysis. The accuracy values obtained by considering data on both resistance and virulence genes are reported respectively in Table 7 for the public dataset and in Table 8 for the Biometec dataset. We observe that, although for some antimicrobial agents (e.g. FOS, SXT, CZA) a higher value of accuracy is observed when data on the virulence genes are also included in the analysis, this is not true for all antimicrobial agents and there are several occurrences (e.g., AZT, COL, MEM, MEM/VAB, and AK) where a slightly lower value of accuracy is obtained. Based on these findings, a definitive answer to the question of whether or not the virulence genes should also be considered cannot be given. However, using data related only to resistance genes generally gives satisfactory performance in terms of values of accuracy that can be obtained.

**Table 7:** Maximum values of accuracy for the public dataset when using only resistance genes or when using both resistance and virulence genes.

Antimicrobial	Maximum values of accuracy	
	Resistance genes	Resistance and virulence genes
AMC	0,936	0,936
FEP	0,949	0,949
CAZ	0,979	0,979
IMI	0,941	0,971
AZT	0,981	0,981
CIP	0,933	0,956
CN	0,953	0,984
COL	0,987	0,987
FOS	0,682	0,758
SXT	0,980	0,960

**Table 8:** Maximum values of accuracy for the Biometec dataset when using only resistance genes or when using both resistance and virulence genes. The results include the six antimicrobial agents common to the public dataset, and the four antimicrobial agents present only in the Biometec dataset.

Antimicrobial	Maximum values of accuracy	
	Resistance genes	Resistance and virulence genes
IMI	0,920	0,952
AZT	0,970	0,966
CN	1,000	NA
COL	0,963	0,957
FOS	0,833	0,900
SXT	0,917	1,000
CZA	0,846	1,000
MEM	0,889	0,870
MEM/VAB	0,920	0,905
AK	0,963	0,926

Lastly, a separate analysis was made for the following antimicrobial agents *Ceftazidime/Avibactam* (CZA), *Meropenem* (MEM), *Meropenem-Vaporbactam* (MEM/VAB) and *Amikacin* (AK), which are present only in the Biometec dataset. These are, in fact, important antimicrobial agents for the clinical treatment of *Klebsiella pneumoniae*.

The results obtained by applying the six ML models of Section Machine learning analysis to these antimicrobial agents are illustrated in Table 9. The values of accuracy range from 0.654 to 0.963. The best performance is obtained with the use of logistic regression, k-nearest neighbors and bagging classifiers.

**Table 9:** Values of accuracy obtained by using different ML classifiers. The highest value(s) accuracy obtained for each antimicrobial agent is (are) highlighted in blue.

ML Classifier	Gaussian Naive Bayes	Logistic regression	k-nearest neighbors	Radius neighbors	Gradient boosting	Bagging
Drug						
**CZA**	0,654	0,846	0,846	0,654	0,731	0,846
**MEM**	0,704	0,815	0,889	0,852	0,889	0,741
**MEM/VAB**	0,920	0,840	0,840	0,760	0,840	0,840
**AK**	0,926	0,963	0,963	0,704	0,963	0,963

## Discussion

In this work we predict antimicrobial resistance in *Klebsiella pneumoniae* using machine learning methods applied to genome sequence data. In particular six machine learning methods were utilized for this purpose. The specific methods include algorithms like Gaussian Naive Bayes, the Logistic regression, the k-nearest neighbors, the radius neighbors, the Gradient Boosting and the Bagging classifier. The performance of the machine learning models was assessed using two distinct datasets, one containing the data collected by the Biometec and one that is a public dataset. These datasets differ from each other in several aspects: the two datasets contain different numbers of *Klebsiella pneumoniae* strains; the origin of the bacteria in each dataset varies, strains are taken from blood, urine and respiratory tract. The datasets also differ for the genes included. Certain genes are associated with specific antimicrobial resistance and thus included as features in the machine learning models. Common metrics for evaluating the performance of machine learning models in this context including accuracy, precision, and recall have been evaluated in our analysis. These parameters provide a quantitative evaluation of the performance of machine learning method in predicting antimicrobial resistance in *Klebsiella pneumoniae* and, ultimately, by comparing the results on the two different databases, offer an indication of which features (genes) are most predictive.

Overall, the six ML models showed good performance in predicting the antibiotic resistance also when only resistance genes are considered, while ignoring the virulence ones. In particular, in the case of the Biometec dataset the highest accuracy value, above 90%, has been obtained when k-nearest neighbors ML model is applied. In general, the prediction of each antimicrobial agent common to the two datasets has a similar value of accuracy, except for the FOS where different accuracy values have been obtained due to the presence/absence of *Fosfomycin*-type gene in the Biometec/public dataset. However, we have not found evidence of a straight correlation between resistance to an antimicrobial and the presence of one or a group of genes, rather the pattern seems far from trivial and, for this reason, unveiling it requires machine learning methods.

The ML models performed well both in the case of the public dataset, which contains a larger number of strains (127) but a smaller number of input genes associated with antimicrobial resistance (only 9 genes resistant), and in the case of the Biometec dataset (57 strains, 34 resistant genes). Despite the limited number of genes in the public dataset, the ML models demonstrated good performance. This suggests that the models were able to effectively learn and generalize patterns of antimicrobial resistance even with a small set of features. The same is also true when the number of available strains is reduced, as we have shown by analyzing not only the Biometec dataset but also a subset of the data contained in the public dataset, consisting of strains originating only from the respiratory tract. Despite the smaller size of these datasets, the ML models still performed well. This indicates the robustness and generalization of the models across different subsets of data, even when dealing with variations in strain origin and dataset size.

We now discuss the limitations of our study that should be considered for accurate interpretation of the overall findings and potential future applications. Firstly, the datasets used in this study are relatively small, which may restrict the ability of the predictive model to generalise correctly when applied to new data. In general, models built on small datasets are susceptible (prone) to overfitting, where the model fits the training data too well but struggles to generalize to new data. Another issue with real datasets is the data imbalance; in our study, one dataset has a higher prevalence of resistant bacterial strains compared to susceptible ones. The imbalance may result in predictive models that do not perform adequately against susceptible strains, reducing the model overall accuracy. Future research could explore methods to manage data imbalance, such as undersampling resistant strains or oversampling susceptible strains.

Furthermore, while the computational times remain very small, we cannot determine in advance (i.e., prior to their application) which of the ML methods has best performance. In fact, the results show that, as the antibiotic changes, the method (or methods) yielding the highest accuracy also changes.

Additionally, the study presented in this paper should be regarded as a preliminary analysis. The results offer an initial indication of the potential of predictive techniques based on genomic data. However, current methods for acquiring such data are relatively slow, posing a significant limitation for their practical application in clinical settings. Timeliness is crucial in managing bacterial infections and deciding on antibiotic therapy, so the time required to obtain genomic data may impede the immediate use of these techniques in everyday clinical practice.

## Conclusion

The use of ML in the study of antibiotic resistance offers several advantages. ML can help process and analyze large amounts of data efficiently, revealing hidden relationships and patterns that may be difficult to detect with conventional methods. An area of interest is the prediction of antimicrobial resistance, as ML can be used to develop predictive models capable of early identifying whether or not a bacterial strain will be resistant to a particular antibiotic. This allows physicians to select the most effective treatment for patients, reducing the inappropriate use of antibiotics and contributing to the fight against antibiotic resistance. It can also contribute to the discovery of new drugs. ML can expedite the discovery of new antibiotics and anti-resistance drugs by analyzing vast databases of chemical compounds and identifying promising molecules for experimentation. Furthermore, the use of ML can be helpful in personalizing treatments. Antibiotic resistance varies among patients and bacterial strains, so it can help personalize antibiotic treatments based on the specific characteristics of the patient and the pathogen, improving treatment efficacy. In summary, the automation offered by ML can reduce the time and resources required to conduct research on antibiotic resistance, accelerating progress in understanding and contrasting this problem.

As a future perspective, to enhance the accuracy of the results, additional analysis could incorporate phenotypic data, including Minimum Inhibitory Concentration (MIC) values. Further investigation is needed in this case, as the biological relationship between a specific mutation’s presence and its phenotypic expression is not always linear, enabling more comprehensive analyses. Also, the inclusion of factors such as geographical location, clinical setting (e.g., hospital-acquired vs. community-acquired infections), previous therapy, antibiotic exposure or other demographic factors could enhance the accuracy and the overall prediction of antimicrobial agents.

## Supporting information

S1 Table Biometec dataset. The dataset contains the genomic data of 57 strains of *Klebsiella pneumoniae*. For each strain, it includes information on the resistance/susceptibility to 15 antimicrobial agents and the presence/absence of 34 resistance genes and 78 virulence genes. In the table, for each strain, a 0 indicates the absence of the specific gene (or susceptibility to the specific antimicrobial agent), while a 1 indicates the presence of the gene (or resistance to the antimicrobial agent).
(ZIP)
